# A Randomized Controlled Trial Evaluating Virologic and Renal Outcomes After Switching from TDF+FTC or 3TC+EFV to TDF/3TC/DTG (TLD) *Versus* DTG+3TC in Virologically Suppressed Thai PWH – A Pilot Study

**DOI:** 10.2174/011570162X384022250910065252

**Published:** 2025-09-29

**Authors:** Sathaporn Kanogtorn, Opass Putcharoen, Samadhi Patamatamkul

**Affiliations:** 1 Department of Medicine, Faculty of Medicine, Suddhavej Hospital, Mahasarakham University, Maha Sarakham, Thailand;; 2 Department of Medicine, Faculty of Medicine, Division of Infectious Diseases, Chulalongkorn University, Bangkok, Thailand;; 3 Emerging Infectious Diseases Clinical Center, Thai Red Cross, Bangkok, Thailand

**Keywords:** Dual antiretroviral therapy, dolutegravir + lamivudine, eGFR, cystatin C, switching regimen, PWH

## Abstract

**Introduction:**

Tenofovir disoproxil fumarate (TDF)/lamivudine (3TC)/dolutegravir (DTG) (TLD) is the preferred first-line therapy for all people living with HIV (PWH) pre WHO 2019 and Thai HIV guidelines. This has prompted switches from TDF + FTC or 3TC + EFV to TLD in Thailand.

**Methods:**

We conducted a randomized trial among virologically suppressed PWH aged ≥18 years on TDF + FTC or 3TC + EFV who were switched to either TLD or DTG + 3TC. The primary outcome was the change in estimated glomerular filtration rate (eGFR) calculated by cystatin C at 24 weeks.

**Results:**

Sixteen participants (eight per group), 69% male with a mean age of 41 years and a median duration of HIV diagnosis of 7.1 years, were enrolled. A greater, though not statistically significant, decline in eGFR was observed in the TLD group. The mean differences were 5.15 mL/min/1.73 m^2^ (95% CI: -12.06 to 22.35; p = 0.532) for cystatin C and 4.01 mL/min/1.73 m^2^ (95% CI: -3.58 to 11.59; p = 0.277) for creatinine. All participants maintained virological suppression. No significant differences were observed in BMI, LDL, or CD4 counts.

**Discussion:**

Although not statistically significant, TLD was associated with a trend toward greater eGFR decline. This finding warrants attention, particularly in patients at risk for renal dysfunction.

**Conclusion:**

Dual therapy with DTG + 3TC may be a preferable switch option over TLD for virologically suppressed PWH with renal safety concerns.

## 1 INTRODUCTION

Combination antiretroviral therapy (cART) has revolutionized the treatment of people living with HIV (PWH), significantly improving survival and quality of life. Triple-drug regimens consisting of two nucleoside reverse transcriptase inhibitors (NRTIs) and one non-nucleoside reverse transcriptase inhibitor (NNRTI) have long been the cornerstone of first-line therapy, particularly in resource-limited settings like Thailand [1]. More recently, the availability of second-generation integrase strand transfer inhibitors (INSTIs) in fixed-dose combinations (FDCs), such as tenofovir disoproxil fumarate (TDF)/lamivudine (3TC)/dolutegravir (DTG) or TLD, has provided a potent and cost-effective alternative [2-4]. Large clinical trials have established TLD as a safe, effective, and well-tolerated regimen, prompting its adoption as the preferred first-line therapy in global and Thai HIV guidelines [1-6]. DTG-based dual therapy, such as DTG + 3TC, has emerged as an alternative to TDF-based regimens, offering comparable virological efficacy with improved renal and metabolic safety profiles [7-9]. The GEMINI trials demonstrated that dual therapy with DTG + 3TC effectively preserved renal function and reduced markers of tubular injury compared to TLD [7]. However, these findings primarily applied to treatment-naïve populations with limited TDF exposure. In contrast, treatment-experienced PWH with prolonged TDF use, particularly those of Asian ethnicity, may have a higher risk of TDF-induced nephrotoxicity due to genetic polymorphism of the multidrug resistance-associated protein 2 (MRP2) or MRP4, and other factors, including lower body mass index (BMI) and subsequent higher tenofovir exposure [10-13]. Given the differences in TDF exposure and baseline characteristics between populations studied in previous trials and the Thai population, this study aimed to compare renal, virological, and metabolic outcomes among Thai PWH who were switched from TDF + FTC or 3TC + EFV to either TLD versus DTG + 3TC. We hypothesized that switching to DTG + 3TC would better preserve eGFR compared to switching to TLD.

## 2 MATERIALS AND METHODS

### 2.1 Study Design and Setting

This open-label, randomized-controlled trial was conducted at two tertiary care centers in Thailand: Suddhavej Hospital, Faculty of Medicine, Mahasarakham University, and King Chulalongkorn Memorial Hospital, Faculty of Medicine, Chulalongkorn University. Recruitment took place from July 26, 2023 to March 31, 2024. Inclusion and exclusion criteria: participants were required to meet the following criteria: confirmed HIV infection by serology or plasma HIV RNA detection, age ≥18 years, virological suppression (HIV RNA <50 copies/mL) within the past 12 months, and current use of TDF + FTC or 3TC + EFV. Exclusion criteria included: 1) Pregnancy; 2) Documented resistance-associated mutations to antiretroviral therapy; 3) eGFR (CKD-EPI) ≤60 mL/min/1.73 m² for participants switching to TLD; 4) Active opportunistic infection or ongoing tuberculosis therapy; 5) Use of contraindicated medications (*e.g*., phenytoin, carbamazepine, midazolam, or St. John’s wort); 6) No creatinine testing within six months prior to switching; 7) Hepatitis B surface antigen positivity.

### 2.2 Interventions

Participants were randomly assigned to switch to either TLD or DTG + 3TC. Both regimens were administered at standard doses, with adjustments made based on creatinine clearance (CrCl) in the dual therapy arm. DTG + 3TC was available as a single FDC or as separate tablets.

### 2.3 Outcomes

The primary outcome was the change in eGFR, calculated using cystatin C and the CKD-EPI equation [14], at 24 weeks (±12 weeks). Secondary outcomes included changes in eGFR calculated by creatinine, virological suppression rates, CD4 counts and percentages, BMI, and LDL levels.

### 2.4 Measurement

Creatinine levels were assessed using an enzymatic creatinine reagent with automated Beckman Coulter AU/DxC AU analyzers (Beckman Coulter, USA) at Mahasarakham University and automated Abbott Alinity c analyzers (Abbott Laboratories, Abbott Park, IL, USA) at King Chulalongkorn Memorial Hospital. Serum cystatin C was quantified using latex-enhanced immunonephelometry (N latex Cystatin C assay) with an Atellica NEPH 630 analyzer (Siemens Healthineers, Marburg, Germany) at Mahasarakham University and by immunoturbidimetry (Tina-quant Cystatin C assay) (Roche Diagnostics, Rotkreuz, Switzerland) at King Chulalongkorn Memorial Hospital.

Plasma HIV-1 RNA levels were measured with the Cobas^®^ 5800 system (Roche, CA, USA) at King Chulalongkorn Memorial Hospital. At Mahasarakham University, plasma HIV-1 RNA samples were processed at a standardized reference laboratory in compliance with the Thai National AIDS program. Low-density lipoprotein (LDL) was analyzed using either fasting or non-fasting samples per each center's protocol.

Adherence was assessed by patient self-report during follow-up visits; no pill count was performed.

## 3 ETHICAL CONSIDERATION

The study was conducted following approval from the Ethics Committee of Mahasarakham University, Mahasarakham, Thailand (No. 296-164/2566), and the Institutional Review Board of the Faculty of Medicine, Chulalongkorn University, Thailand (No. 0349/66). The trial is registered with the Thai Clinical Trials Registry (TCTR20221117001). Written informed consent was obtained from all participants at the time of recruitment. This trial was conducted and reported in accordance with the Consolidated Standards of Reporting Trials (CONSORT) 2010 guidelines [15].

### 3.1 Statistical Analysis

A mixed-effects model was used to evaluate changes in eGFR between groups, as well as key secondary outcomes such as BMI, metabolic profiles, and CD4 counts and percentages. Mixed-effects models were used to compare changes between the two groups over time, as this approach appropriately accounts for repeated measures and individual-level variability, and is more robust to missing data and unbalanced follow-up. Additionally, the model assumptions were assessed through visual inspection of diagnostic plots. The QQ plot of residuals indicated approximate normality, with points generally aligning along the diagonal. The residual plot (residuals *vs*. predicted values) and homoscedasticity plot (absolute residuals *vs*. predicted values) showed no discernible patterns, suggesting linearity and constant variance of residuals. Although the small sample size limits formal testing of model assumptions, the fixed-effect estimates from linear mixed-effects models are generally robust to minor violations of these assumptions.

Paired t-tests were performed to assess within-group changes and were only used after confirming normality of the data within each group using the Shapiro–Wilk test. Virological suppression (HIV viral load below 50 copies per milliliter) at 24 weeks, including a 12-week window on either side, was analyzed using the chi-square test or Fisher’s exact test. Statistical analyses and graphical presentations were performed using Statistical Package for the Social Sciences (SPSS) version 28 (IBM Corp., Armonk, NY, USA) accessed *via* Chulalongkorn University, and GraphPad Prism version 10 (GraphPad Software, Inc., San Diego, CA, USA). A two-sided *P*-value of ≤ 0.05 was considered statistically significant.

## 4 RESULTS

### 4.1 Baseline Characteristics

Among 149 participants assessed for eligibility, 16 participants were randomized and completed the 24-week follow-up. Details of the recruitment process are provided in Fig. (**1**). The mean age was 41.32 years, with 69% of participants being male. The average time since HIV diagnosis was 7.1 years, and the mean duration of TDF exposure before switching was 6.2 years. Baseline characteristics, including eGFR, BMI, and metabolic profiles, were similar between the groups, although the DTG + 3TC group showed a slightly higher baseline BMI (23.55 kg/m² *vs*. 22.87 kg/m², *p* = 0.09). No participants had active opportunistic infections or were receiving nephrotoxic drugs at enrollment (Table **1**).

### 4.2 Renal Outcomes

**Fig. (2):**
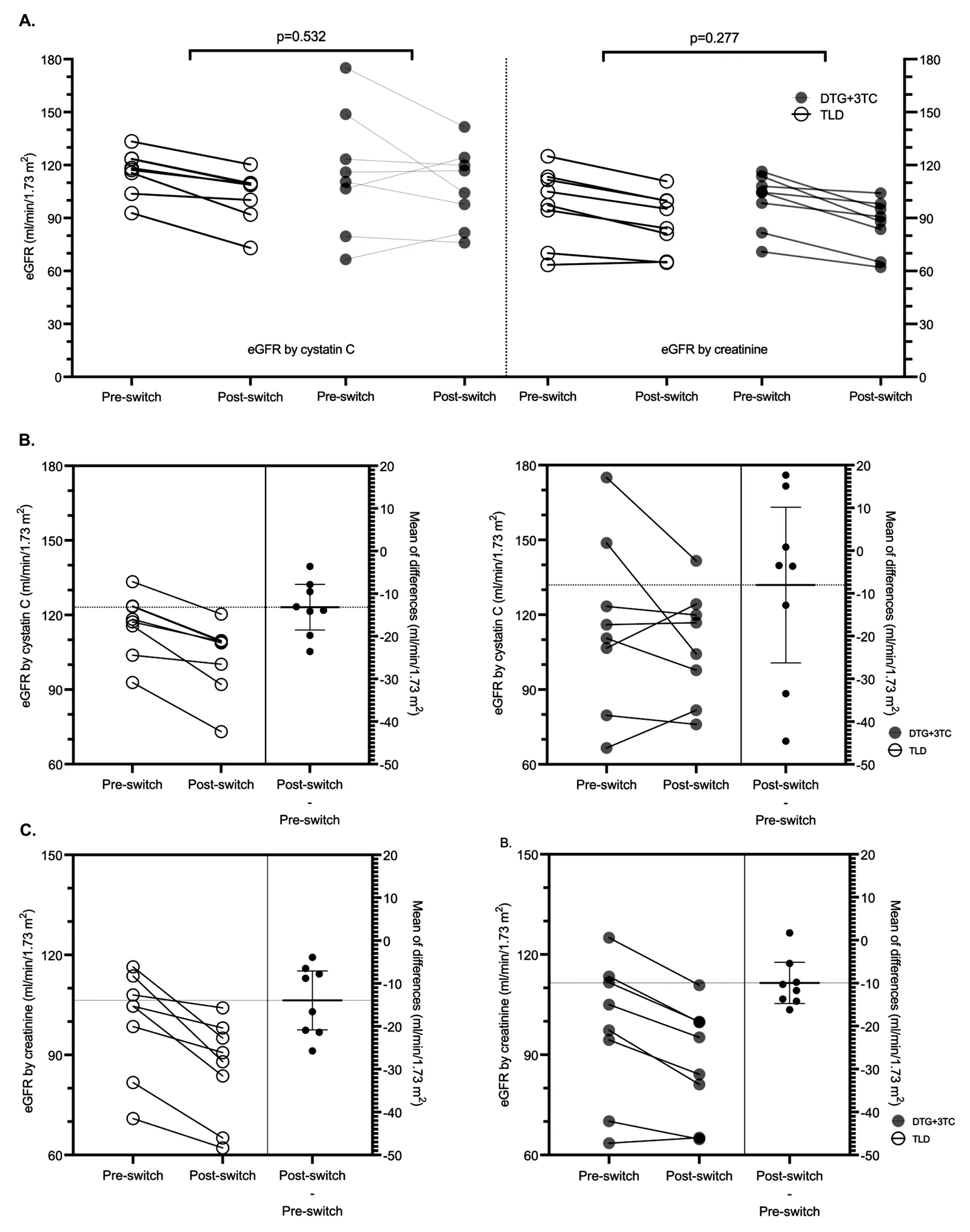
(**A**) Comparison of eGFR changes over time, with values calculated using cystatin C shown on the left side and creatinine on the right side. (**B**) Left: Change in eGFR by cystatin C from pre-switch to post-switch in the TLD group. Right: Change in eGFR by cystatin C from pre-switch to post-switch in the DTG + 3TC group. (**C**) Left: Change in eGFR by creatinine from pre-switch to post-switch in the TLD group. Right: Change in eGFR by creatinine from pre-switch to post-switch in the DTG + 3TC group.

The mean reduction in eGFR calculated using cystatin C was greater in the TLD group compared to the DTG + 3TC group (-13.19 *vs*. -8.04 mL/min/1.73 m²) (Fig. **2A**, right column). This corresponded to a mean difference in eGFR change between the two groups of 5.15 mL/min/1.73 m² (95% CI -12.06 to 22.35) based on mixed-effects model analysis (*p* = 0.532). However, within-group analysis revealed a significant eGFR decline in the TLD group (mean change: -13.19 mL/min/1.73 m², 95% CI -18.53 to -7.84, *p* < 0.001), whereas the decline in the DTG + 3TC group was not significant (-8.04 mL/min/1.73 m², 95% CI -26.24 to 10.16, *p* = 0.331) (Fig. **2B**). Similarly, eGFR calculated using creatinine declined more in the TLD group than in the DTG + 3TC group (-13.95 *vs*. -9.95 mL/min/1.73 m², *p* = 0.277) (Fig. **2A**). This corresponded to a 4.01 (95% CI -3.58 to 11.59) mL/min/1.73 m^2^ difference of mean eGFR change between the two groups. Both groups experienced significant within-group reductions (TLD: -13.95 mL/min/1.73 m², 95% CI -20.81 to -7.1, *p* = 0.002; DTG + 3TC: -9.95 mL/min/1.73 m², 95% CI -14.75 to -5.15, *p* = 0.002) (Fig. **2C**).

The scatter dot plot illustrates the individual changes in eGFR for each participant (Figs. **2A**-**C**). Scatter dot plots on the left sub column depict the differences in eGFR change within each group (Figs. **2B**, **C**). The error bars denote the 95% confidence interval of the mean change, with the thick central line indicating the mean value.

### 4.3 Virological and Metabolic Outcomes

**Fig. (3):**
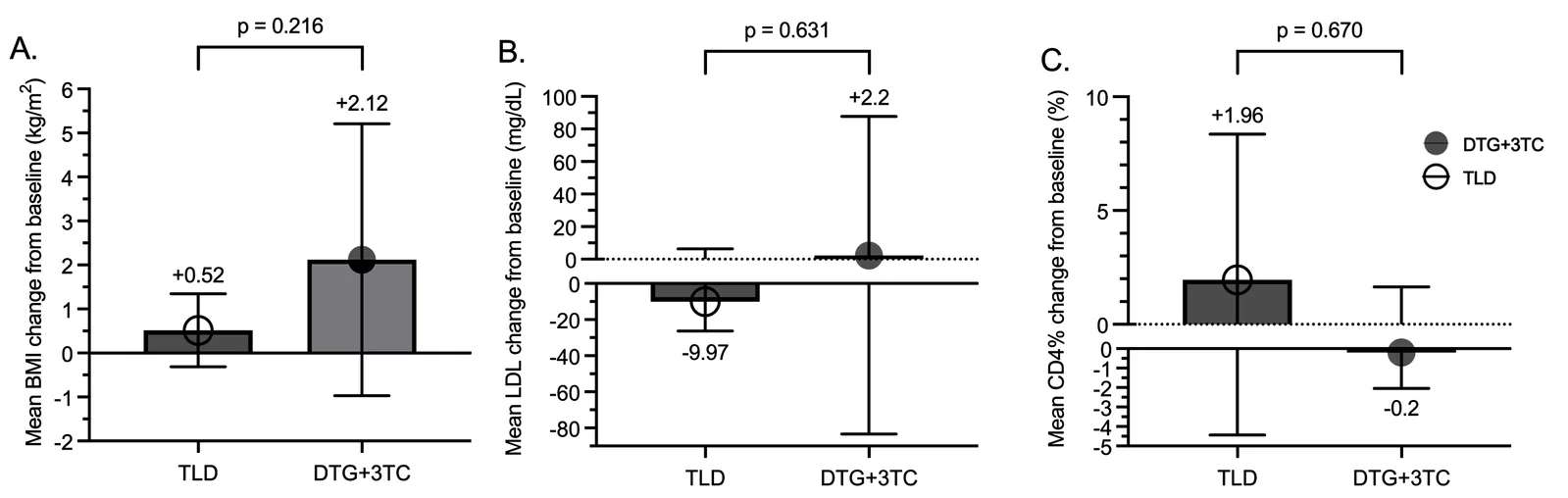
Comparison of mean changes from baseline to the second follow-up for: (**A**) Body Mass Index (BMI), (**B**) Low-Density Lipoprotein (LDL), and (**C**) CD4 percentage (CD4%) between the TLD and DTG + 3TC groups.

Virological suppression rates were maintained in 100% of participants in both groups. No significant differences in metabolic parameters, including LDL and BMI changes, were observed between the groups. However, BMI tended to increase more in the DTG + 3TC group compared to the TLD group, with a mean difference of 1.6 kg/m² (95% CI -1.06 to 4.27, *p* = 0.216) (Fig. **3A**). Similarly, LDL levels increased more in the DTG + 3TC group than in the TLD group (mean difference: 11.72 mg/dL, 95% CI -37.98 to 61.41, *p* = 0.631) (Fig. **3B**). CD4 counts and percentages showed no significant differences between groups. The mean difference in CD4 count changes was -119.2 cells/mm³ (95% CI -262.1 to 23.77, *p* = 0.093), while the mean difference in CD4 percentage changes was -1.4% (95% CI -8.48 to 5.69, *p* = 0.670) (Fig. **3C**). Details of within-group changes in eGFR by cystatin C, creatinine, CD4 count, BMI, and LDL—calculated using paired t-tests—are presented in Table **2**.

The error bars represent the 95% confidence interval of the mean changes, calculated using paired T-tests within each group.

Safety outcomes revealed no severe adverse events, and no participants discontinued their assigned regimens.

## 5 DISCUSSION

This pilot randomized trial suggests that switching to DTG + 3TC may offer better renal preservation, as indicated by cystatin C-based eGFR, compared to switching to TLD. These findings align with results from the GEMINI trials, where DTG + 3TC demonstrated a superior renal safety profile relative to TLD [8]. However, the small sample size and the inclusion of outliers with elevated baseline cystatin C eGFR likely impacted statistical significance. Sensitivity analysis excluding 2 outliers with baseline eGFR by cystatin C >140 mL/min/1.73 m² revealed a significant difference favoring DTG + 3TC. This analysis showed a markedly greater eGFR reduction by cystatin C in the TLD group compared to DTG + 3TC, with a mean difference of 15.47 mL/min/1.73 m² (95% CI 4.81–26.13; *p* = 0.008), underscoring the potential renal advantages of dual therapy for individuals with prolonged TDF exposure.

Asian populations, such as the Thai cohort in this study, are believed to have heightened susceptibility to TDF-induced nephrotoxicity due to genetic polymorphisms in ABCC2 and ABCC4, which may increase tenofovir accumulation in renal proximal tubular cells [10-13]. Lower BMI and extended TDF exposure are additional risk factors for proximal tubular dysfunction and eGFR decline [16]. While TLD remains highly effective for virological suppression, the observed renal benefits with DTG + 3TC suggest it may be a more suitable option for individuals at heightened risk for nephrotoxicity. This is crucial because early discontinuation of TDF in cases of TDF-induced nephrotoxicity leads to significantly higher rates of renal function recovery, particularly in the improvement of CrCl and the reduction of tubular electrolyte loss [17].

Virological suppression remained consistently high in both groups, corroborating the efficacy of DTG + 3TC and TLD as observed in larger trials such as GEMINI-1/2, SALSA, and TANGO [7-9]. Moreover, multiple studies comparing dual therapy (DTG/3TC or DTG + 3TC) with combination triple therapy have consistently demonstrated comparable efficacy, safety, and tolerability over extended follow-up periods. These findings support the generalizability of dual therapy as a viable option, particularly for individuals at risk for TDF-induced proximal renal tubulopathy [18-20].

In terms of metabolic outcomes, BMI and LDL levels tended to increase more in the DTG + 3TC group, albeit without statistical significance. Notably, LDL levels have been shown to decrease in some studies comparing DTG + 3TC with EFV- or boosted protease inhibitor-based regimens, where a higher baseline risk of hyperlipidemia was anticipated [21]. However, in comparisons between DTG + 3TC and TLD, the GEMINI-1/2 trials observed a trend toward higher LDL levels in the DTG + 3TC group [7]. It is possible that with longer follow-up, LDL levels in the DTG + 3TC group may demonstrate a statistically significant increase compared to those in the TLD group. However, LDL elevation, a modifiable cardiovascular risk factor, should be addressed with lifestyle modifications and lipid-lowering treatments, as demonstrated by the REPRIEVE trial [22].

Regarding weight gain in DTG-based therapy, previous studies—including SALSA and TANGO—have suggested that discontinuing TDF and EFV, both of which are associated with weight-suppressive effects, may help explain this trend [8-9]. This pattern was also observed in our trial, where a slightly more pronounced increase in BMI levels was seen in the dual therapy group. However, it remains unclear whether BMI will increase more significantly in the DTG + 3TC group compared to TLD with longer follow-up among Thai PWH, as has been commonly observed in other ethnic groups receiving DTG-based regimens [23-26]. In a cohort of treatment-naïve Thai PWH comparing EFV-based and DTG-based regimens, no significant weight gain was observed in the DTG group, despite EFV’s known weight-suppressive effect [27]. Body weight changes in PWH are likely multifactorial, influenced by genetics, environmental exposures, lifestyle factors, antiretroviral regimens, opportunistic infections, and comor-bidities. Therefore, individualization of care—taking these factors into account—is essential when considering the risk of weight gain associated with DTG-based regimens [27].

There are several limitations to our study. First, the sample size was small, as it was pre-specified as a pilot study; therefore, the generalizability of the findings is limited. Second, the short follow-up duration may have favored the null hypothesis, resulting in an insignificant difference in eGFR change. TDF-induced proximal renal tubulopathy may recover gradually and can take months to several years after TDF withdrawal to show meaningful improvement [28, 29]. A similar limitation applies to metabolic outcomes, such as LDL cholesterol levels, where the observed difference between groups was not statistically significant. However, a longer follow-up period may reveal a more pronounced increase, particularly given the upward trend in the dual therapy group [7-9]. Fourth, there was a slight imbalance in follow-up duration between the two groups, as not all participants strictly adhered to scheduled visits. This may have introduced minor inaccuracies in comparing eGFR changes, especially since participants in the dual therapy group had earlier follow-up, and any potential eGFR improvement may not yet have been evident. Lastly, adherence to statins or cART was assessed only through patient self-report at the follow-up visit. Inconsistent adherence may have introduced bias in some parameters, particularly with respect to statin use and LDL measurements.

## CONCLUSION

This pilot randomized study suggests that switching to DTG + 3TC may offer renal preservation benefits compared to TLD, particularly when cystatin C is used to assess kidney function. While the observed difference was more evident in sensitivity analysis, the findings should be interpreted cautiously due to the small sample size. These preliminary results support the need for larger studies to better evaluate the renal safety of dual therapy, especially in populations with prolonged TDF exposure.

## AUTHORS’ CONTRIBUTION

The authors confirm their contribution to this paper as follows: O. P.: Conceptualization; Writing – review and editing. S. K.: Investigation; Writing – review and editing. S. P.: Conceptualization; Writing – original draft; Writing – review and editing.

## Figures and Tables

**Fig. (1):**
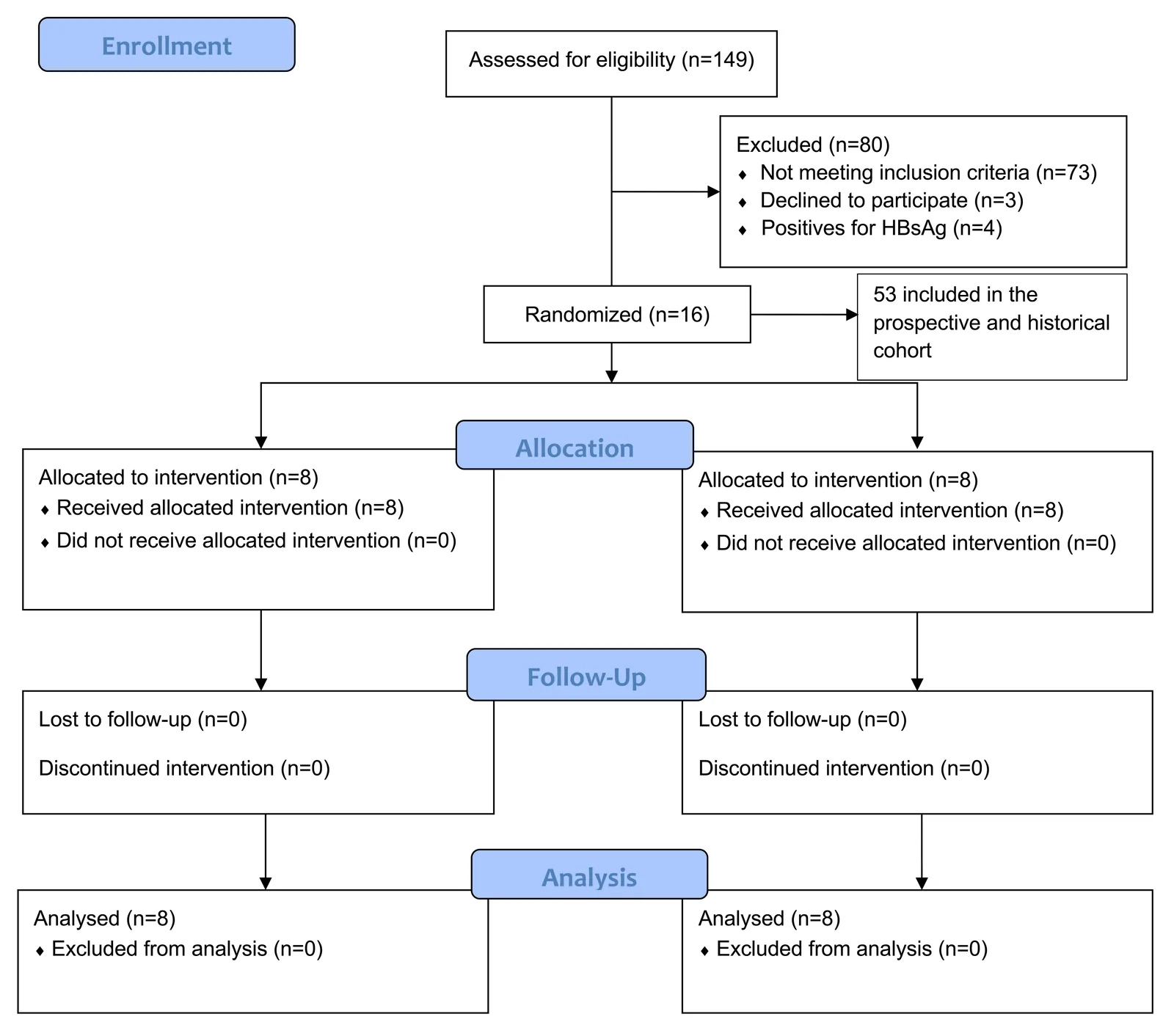
Flow of case recruitment.

**Table 1:** Baseline characteristics and pre-switch laboratory markers in the two groups.

Baseline Characteristics	TLD (n = 8)	DTG+3TC (n = 8)
Age, years (± SD)	35.88 ± 8.36	46.75 ± 12.08
Gender	-	-
Male, n (%)	6 (75)	5 (62.5)
Body weight, kg (± SD)	60.74 ± 8.37	71.36 ± 12.92
Body mass index, kg/m^2^ (± SD)	21.62 ± 3.29	25.47 ± 1.87
Duration from HIV diagnosis to switching, years (± SD)	5.87 ± 2.78	8.25 ± 4.47
Nadir CD4, cells/mm^3^ (± SD)	421.75 ± 257.54	190.75 ± 145.88
Pre-switch CD4, cells/mm^3^ (± SD)	491.75 ± 190.35	596.13 ± 271.76
Pre-switch CD4, % (± SD)	22.8 ± 9.69	26.86 ± 10.16
Comorbidities, n (%)	-	-
Diabetes mellitus	0 (0)	2 (25)
Chronic kidney disease	0 (0)	0 (0)
Hypertension	1 (12.5)	1 (12.5)
Hypo/hyperthyroidism	0 (0)	0 (0)
Previous opportunistic infection, n (%)	-	-
Tuberculosis	1 (12.5)	1 (12.5)
Cryptococcosis	0 (0)	0 (0)
Other endemic dimorphic fungi	0 (0)	0 (0)
Pneumocystis pneumonia	0 (0)	1 (12.5)
CMV retinitis	0 (0)	0 (0)
Current medication	-	-
ACEI or ARB	0 (0)	1 (12.5)
CCB	0 (0)	1 (12.5)
Diuretics†	0 (0)	0 (0)
SGLT-2 inhibitors	1 (12.5)	0 (0)
Statin	1 (12.5)	4 (50)
Duration of TDF prior to switch, months (± SD)	4.86 ± 2.08	7.51 ± 3.18
Pre-switch creatinine, mg/dL (± SD)	0.93 ± 0.2	0.84 ± 0.13
Pre-switch eGFR by CKD-EPI	-	-
(mL/min/1.73 m^2^) (± SD)	99.8 ± 15.79	97.54 ± 21.31
Pre-switch cystatin C, mg/dL (± SD)	0.75 ± 0.09	0.72 ± 0.23
Pre-switch eGFR by cystatin C by CKD-EPI (mL/min/1.73 m^2^) (± SD)	116.02 ± 12.58	115.82 ± 34.84
LDL, mg/dL (± SD)	122.41 ± 21.88	123.63 ± 43.99
Time to the second follow-up, months (± SD)	8.34 ± 0.94	5.86 ± 2.69

**Note:** †Diuretics include any of the following medications: hydrochlorothiazide (HCTZ), furosemide, spironolactone, finerenone, tolvaptan, or torsemide. **Abbreviations:** TLD = tenofovir disoproxil fumarate + lamivudine + dolutegravir (TDF+3TC+DTG); TDF = tenofovir disoproxil fumarate; 3TC = lamivudine; DTG = dolutegravir; ACEI = angiotensin-converting enzyme inhibitor; ARB = angiotensin receptor blocker; LDL = low-density lipoprotein; PRT = proximal renal tubulopathy.

**Table 2:** Within-group changes in eGFR (cystatin C and creatinine), CD4 count, BMI, and LDL from baseline to follow-up (paired t-test analysis).

**At the 2^nd^ Visit**	**TLD (n = 8)**	**DTG+3TC (n = 8)**	***p*-value from Mixed-Effects Model**
Mean eGFR change by cystatin C, mL/min/1.73 m^2^ (95% CI)	-13.19 (95% CI -18.53 to -7.84) *p*<0.001	-8.04 (95% CI -26.24 to 10.16) *p*=0.331	0.532
Mean eGFR change by creatinine, mL/min/1.73 m^2^ (95% CI)	-13.95 (95% CI -20.81 to -7.1) *p*=0.002	-9.95 (95% CI -14.75 to -5.15) *p*=0.002	0.277
Change of CD4+ number, cells/mm^3^ (95% CI)	-33.71 (95% CI -72.35 to 139.8) *p*=0.466	-101.6 (95% CI -226.0 to 22.80) *p*=0.086	0.093
Change of CD4+ percentage, % (95% CI)	1.96 (95% CI -4.436 to 8.35) *p*=0.482	-0.02 (95% CI -2.04 to 1.64) *p*=0.778	0.670
Changes of BMI, kg/m^2^ (95% CI)	0.52 (95% CI -0.31 to 1.34) *p*=0.185	2.12 (95% CI -0.97 to 5.21) *p*=0.145	0.216
Changes of LDL, mg/dL (95% CI)	-9.97 (95% CI -26.36 to 6.42) *p*=0.187	2.2 (95% CI -83.36 to 87.76) *p*=0.947	0.631

**Note:** P-values within each box represent results from within-group analysis using paired T-tests, while the rightmost column shows between-group P-values from a mixed-effects model. Abbreviations: eGFR = estimated glomerular filtration rate; CI = confidence interval; CD4 = cluster of differentiation 4; BMI = body mass index; LDL = low-density lipoprotein; TLD = tenofovir disoproxil fumarate + lamivudine + dolutegravir; DTG + 3TC = dolutegravir + lamivudine.

## Data Availability

The data supporting this study are available from the corresponding author upon request.

## LIST OF ABBREVIATIONS

cART: Combination Antiretroviral Therapy
PWH: People Living with HIV
NRTIs: Nucleoside Reverse Transcriptase Inhibitors
FDCs: Fixed-dose Combinations
INSTIs: Integrase Strand Transfer Inhibitors
TDF: Tenofovir Disoproxil Fumarate
MRP2: Multidrug Resistance-associated Protein 2

## STANDARDS OF REPORTING

CONSORT guidelines were followed.

## References

[1] (2019). Update of recommendations on first- and second-line antiretroviral regimens.

[2] Venter W. D. F., Moorhouse M., Sokhela S. (2019). Dolutegravir plus two different prodrugs of tenofovir to treat HIV. N Engl J Med.

[3] (2021). The Thai National Guidelines on HIV/AIDS Treatment and Prevention. https://www.thaiaidssociety.org/wp-content/uploads/2023/03/HIV-AIDS-Guideline-2564_2565_ED2.pdf.

[4] Goh O. Q., Colby D. J., Pinyakorn S. (2019). Switch to dolutegravir is well tolerated in Thais with HIV infection. J Int AIDS Soc.

[5] Meyer-Rath G, Jamieson L, Moultrie H (2021). The cost-effectiveness of transitioning to dolutegravir-based regimens for first-line antiretroviral therapy in South Africa. Lancet HIV.

[6] (2020). The Thai National Guidelines on HIV/AIDS Diagnosis, Treatment and Prevention 2020/2021. https://www.thaiaidssociety.org/wp-content/uploads/2022/02/Thailand-National-Guidelines-on-HIV-AIDS-Diagnosis-Treatment-and-Prevention-2020-2021.pdf.

[7] Cahn P, Madero J. S., Arribas J. R. (2020). Durable efficacy of dolutegravir plus lamivudine in antiretroviral treatment-naïve adults with HIV-1 infection: 96-week results from the GEMINI-1 and GEMINI-2 randomized clinical trials. J Acquir Immune Defic Syndr.

[8] van Wyk J, Ajana F, Bisshop F (2020). Efficacy and safety of switching to dolutegravir/lamivudine fixed-dose 2-drug regimen vs continuing a tenofovir alafenamide-based 3- or 4-drug regimen for maintenance of virologic suppression in adults living with human immunodefi-ciency virus type 1: Phase 3, randomized, noninferiority TANGO study. Clin Infect Dis.

[9] Llibre J. M., Brites C., Cheng C. Y. (2023). Efficacy and safety of switching to the 2-drug regimen dolutegravir/lamivudine versus continuing a 3- or 4-drug regimen for maintaining virologic suppression in adults living with HIV-1: Week 48 results from the phase 3, noninferiority SALSA randomized trial. Clin Infect Dis.

[10] Manosuthi W, Sukasem C, Thongyen S, Nilkamhang S, Sungkanuparph S (2014). ABCC2*1C and plasma tenofovir concentration are correlated to decreased glomerular filtration rate in patients receiving a tenofovir-containing antiretroviral regimen. J Antimicrob Chemother.

[11] Nishijima T, Komatsu H, Higasa K (2012). Single nucleotide polymorphisms in ABCC2 associate with tenofovir-induced kidney tubular dysfunction in Japanese patients with HIV-1 infection: A pharmacogenetic study. Clin Infect Dis.

[12] Likanonsakul S, Suntisuklappon B, Nitiyanontakij R (2016). A single-nucleotide polymorphism in ABCC4 is associated with tenofovir-related β2-microglobulinuria in Thai patients with HIV-1 infection. PLoS One.

[13] Rodríguez-Nóvoa S, Labarga P, D’Avolio A (2010). Impairment in kidney tubular function in patients receiving tenofovir is associated with higher tenofovir plasma concentrations. AIDS.

[14] Levey A. S., Stevens L. A., Schmid C. H. (2009). A new equation to estimate glomerular filtration rate. Ann Intern Med.

[15] Schulz K. F., Altman D. G., Moher D. (2010). CONSORT 2010 Statement: Updated guidelines for reporting parallel group randomised trials. BMJ.

[16] Ezinga M, Wetzels JFM., Bosch MEW., van der Ven AJAM., Burger D. M. (2014). Long-term treatment with tenofovir: Prevalence of kidney tubular dysfunction and its association with tenofovir plasma concentration. Antivir Ther.

[17] Patamatamkul S, Songumpai N, Payoong P, Katavetin P, Putcharoen O (2022). Early switching of tenofovir disoproxil fumarate (TDF) in HIV-infected patients with TDF-induced nephrotoxicity: A prospective study. HIV Res Clin Pract.

[18] Fairhead C, Levi J, Ramesh N, Hill A (2024). Results from systematic review and meta-analysis of the efficacy and safety of two-drug versus three-drug (tenofovir/lamivudine/dolutegravir; TLD) regimens for HIV treatment. Clin Infect Dis.

[19] Punekar Y. S., Parks D., Joshi M. (2021). Effectiveness and safety of dolutegravir two‐drug regimens in virologically suppressed people living with HIV: A systematic literature review and meta‐analysis of real‐world evidence. HIV Med.

[20] Russo A, Martini S, Pisaturo M (2024). Efficacy and tolerability of INI-based 2-drug regimens in virologically suppressed persons living with HIV: A systematic review and meta-analysis. Infect Chemother.

[21] Letang E, Lo J, Milinkovic A, Maggiolo F, Di Giambenedetto S, Mussini C (2022). Lipid changes in real-world studies with the 2-drug regimen dolutegravir and lamivudine (DTG + 3TC) in people with HIV-1: A systematic literature review. Presented at:. HIV Drug Therapy Glasgow UK, 2022 Oct 23–26..

[22] Grinspoon S. K., Fitch K. V., Zanni M. V. (2023). Pitavastatin to prevent cardiovascular disease in HIV infection. N Engl J Med.

[23] Hirigo A. T., Yilma D., Astatkie A., Debebe Z. (2023). Effect of dolutegravir-based first-line antiretroviral therapy on weight and body mass index among adult people living with HIV on follow up at health facilities in Hawassa city administration, Southern Ethiopia: A retrospective co-hort study. Ann Med.

[24] Thivalapill N, Simelane T, Mthethwa N (2021). Transition to dolutegravir is associated with an increase in the rate of body mass index change in a cohort of virally suppressed adolescents. Clin Infect Dis.

[25] Suárez-García I, Alejos B, Hernando V (2023). Effectiveness and tolerability of dolutegravir/lamivudine for the treatment of HIV-1 infection in clinical practice. J Antimicrob Chemother.

[26] Kanters S, Renaud F, Rangaraj A (2022). Evidence synthesis evaluating body weight gain among people treating HIV with antiretroviral ther-apy - A systematic literature review and network meta-analysis. E Clin Med.

[27] Setpakdee R, Kiertiburanakul S (2025). Effect of dolutegravir on weight changes and lipid profile compared with efavirenz in people living with HIV: A retrospective cohort study. J Infect Public Health.

[28] Jose S, Hamzah L, Campbell LJ (2014). Incomplete reversibility of estimated glomerular filtration rate decline following tenofovir disoproxil fumarate exposure. J Infect Dis.

[29] Patamatamkul S, Kanogtorn S, Putcharoen O (2025). P 580. A randomized controlled trial evaluating virologic and renal outcomes after switching from TDF/FTC/EFV to TDF/3TC/DTG (TLD) versus DTG+3TC in virologically suppressed Thai PWH– a pilot study. Open Forum Infect Dis.

